# Immune Function and Micronutrient Requirements Change over the Life Course

**DOI:** 10.3390/nu10101531

**Published:** 2018-10-17

**Authors:** Silvia Maggini, Adeline Pierre, Philip C. Calder

**Affiliations:** 1 Bayer Consumer Care AG, 4002 Basel, Switzerland; adeline.pierre@bayer.com; 2 Human Development & Health, Faculty of Medicine, University of Southampton, Southampton SO16 6YD, UK; P.C.Calder@soton.ac.uk; 3 NIHR Southampton Biomedical Research Centre, University Hospital Southampton NHS Foundation Trust and University of Southampton, Southampton SO16 6YD, UK

**Keywords:** adults, age-related immunity, deficiency, elderly, immunosenescence, infants, infection, micronutrients, older people

## Abstract

As humans age, the risk and severity of infections vary in line with immune competence according to how the immune system develops, matures, and declines. Several factors influence the immune system and its competence, including nutrition. A bidirectional relationship among nutrition, infection and immunity exists: changes in one component affect the others. For example, distinct immune features present during each life stage may affect the type, prevalence, and severity of infections, while poor nutrition can compromise immune function and increase infection risk. Various micronutrients are essential for immunocompetence, particularly vitamins A, C, D, E, B2, B6, and B12, folic acid, iron, selenium, and zinc. Micronutrient deficiencies are a recognized global public health issue, and poor nutritional status predisposes to certain infections. Immune function may be improved by restoring deficient micronutrients to recommended levels, thereby increasing resistance to infection and supporting faster recovery when infected. Diet alone may be insufficient and tailored micronutrient supplementation based on specific age-related needs necessary. This review looks at immune considerations specific to each life stage, the consequent risk of infection, micronutrient requirements and deficiencies exhibited over the life course, and the available evidence regarding the effects of micronutrient supplementation on immune function and infection.

## 1. Introduction

**Figure 1:**
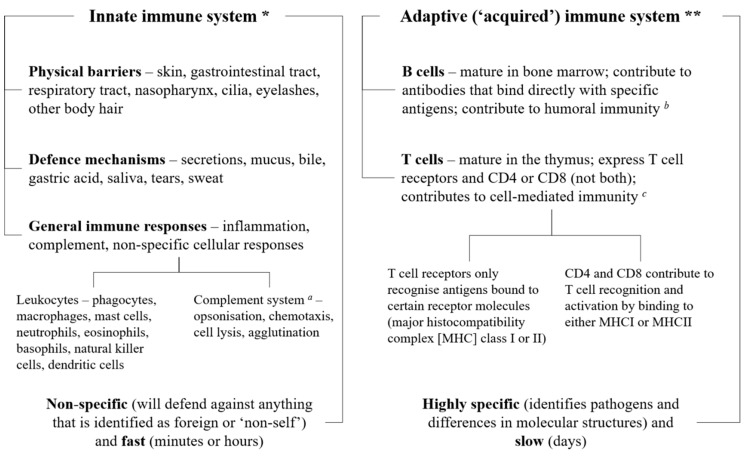
Simple overview of the immune system. The three layers of the immune system (physical and biochemical barriers; cells such as monocytes, granulocytes, lymphocytes, and B and T cells; and antibodies or immunoglobulins) work together to protect the body against pathogens, utilizing the innate and adaptive defense mechanisms. All three layers are involved in the innate and immune systems. * The innate immune system comprises anatomical and biochemical barriers and an unspecific cellular response mediated mainly by monocytes, neutrophils, natural killer cells and dendritic cells; these work together to fight off pathogens before they can start an active infection. ** The adaptive immune system involves an antigen-specific response mediated by T and B lymphocytes that is activated by exposure to pathogens; this works with the innate immune system to reduce the severity of infection. *^a^* The complement system can work with both the innate and adaptive immune systems; *^b^* i.e., immunity from serum antibodies produced by plasma cells; *^c^* i.e., an immune response that does not involve antibodies, but responds to any cells that display aberrant major histocompatibility complex (MHC) markers, such as cells invaded by pathogens.

The immune system, which is integrated into all physiological systems, protects the body against infections and other external and internal insults by utilizing three distinct layers, depending on the nature of the threat: physical (e.g., skin, epithelial lining of the gastrointestinal and respiratory tracts) and biochemical barriers (e.g., secretions, mucus, and gastric acid), numerous different immune cells (e.g., granulocytes, CD4 or CD8 T and B cells), and antibodies (i.e., immunoglobulins). The first line of defense is innate immunity, which combines physical and biochemical barriers with a non-specific, leukocyte-mediated cellular response to defend against pathogens [1]. If the pathogen manages to avoid these innate defenses, a more complex, adaptive, antigen-specific response is triggered, mediated by T and B lymphocytes, which produces antibodies to target and destroy the pathogen (Figure 1) [1]. Both systems also protect against native cells that may be harmful, such as cancerous or precancerous cells [2].

As humans age, the immune system evolves from the immature and developing immune responses in infants and children, through to immune function that is potentially optimal in adolescents and young adults, followed by a gradual decline in immunity (particularly adaptive processes) in older people [1]. Age-related changes are compounded by certain lifestyle factors (e.g., diet, environmental factors, and oxidative stress) specific to each life stage that can influence and modify, in some cases suppressing, immune function. Accordingly, the risk and severity of infections such as the common cold and influenza (the most common illnesses in humans [3]), pneumonia and diarrheal infections also vary over a lifetime.

Optimal immune function is dependent on a healthy immune system. In turn, adequate nutrition is crucial to ensure a good supply of the energy sources, macronutrients and micronutrients required for the development, maintenance and expression of the immune response [3]. Micronutrients have vital roles throughout the immune system that are independent of life stage (Table 1), and it has been determined that those most needed to sustain immunocompetence include vitamins A, C, D, E, B2, B6 and B12, folic acid, beta carotene, iron, selenium, and zinc [4]. There is a bidirectional interaction among nutrition, infection and immunity: the immune response is compromised when nutrition is poor, predisposing individuals to infections, and a poor nutritional state may be exacerbated by the immune response itself to an infection [5]. It is clear that optimal immunocompetence depends upon nutritional status [6]. It is recognized that micronutrient deficiencies and suboptimal intakes are common worldwide [7], and certain micronutrients may be more likely to be insufficient at different stages of the life course. This can affect the risk and severity of infection, and in fact an individual’s nutritional status can predict the clinical course and outcome of certain infections such as diarrhea, pneumonia and measles [4]. Resistance to infection may be enhanced by adding the deficient nutrient back into the diet and restoring immune function [4]. However, it is not always possible to achieve good nutritional status via the diet alone. In developing countries, for example, it may be difficult to find an adequate and varied supply of food. Even in industrialized nations, where it may be presumed that healthy, nutritious food is easier to obtain, social, economic, educational, ethnic and cultural backgrounds influence the diet and may adversely affect an individual’s micronutrient status [8].

This review looks at life-stage-specific immunity, risk of infection and micronutrient requirements, from the perspective of industrialized countries where possible. The aim is to highlight the role of tailored supplementation in restoring micronutrients to recommended levels and better supporting immune needs that are specific to each life stage.

## 2. The Immune System

### 2.1. Infants and Children

Prior to birth, babies lack significant antigenic exposure and so have not yet acquired immunological memory and their adaptive immunity is not fully developed [5,10,11,19]. Therefore, immune protection from pathogens such as bacteria and viruses immediately after birth relies on two primary methods of defense, passive immunity and innate immunity. Passive immunity is where maternal antibodies (antigen-specific immunoglobulins) are passed via the placenta before birth, and in maternal colostrum and milk after birth [10]. The primary immunoglobulin (Ig) in human maternal milk is IgA (which plays a crucial role in immune function at mucosal surfaces), but IgG (which provides the majority of antibody-based immunity against invading pathogens) and IgM (which eliminates pathogens in the early stages of B cell-mediated or humoral immunity before there is sufficient IgG) are also present in smaller amounts [20]. Levels of all immunoglobulins in maternal milk decrease in the days following birth [20], and babies and children are more susceptible to infections until they are able to produce sufficient antibodies by themselves. Maternal milk is a rich source of cells and compounds with immunological properties, depending on the stage of lactation, and may facilitate immune development and maturation in infants [21,22]. These include leukocytes (neutrophils, macrophages), cytokines, complement, and long-chain polyunsaturated fatty acids, which variously have antimicrobial, tolerance/priming, immune development, and anti-inflammatory properties [21,22].

The baby’s innate immune system is essential to defend against pathogens [10]. The innate system is still functionally immature at birth, to allow the fetus to tolerate non-shared maternal antigens, but also so that it is not constantly triggered by the considerable amount of stress and remodeling that take place during development [19]. The neonatal innate immune system comprises different protective cell populations compared with adults, as well as qualitative differences in the responses by shared cell populations [23]. For example, innate immune cells such as monocytes and dendritic cells produce less of the bioactive form of interleukin (IL)-12 and type 1 interferon in newborns compared with adults, but similar or higher amounts of other interleukins (e.g., IL-6, IL-10, and IL-23) when stimulated by the same pathogen [24]. Neonatal cells are also less able to produce multiple cytokines in response to pathogenic stimulation [24]. Concentrations of NK cells are at their lowest in infants compared with other life-stages [25]. Furthermore, serum concentrations of almost all circulating components of the complement system are much lower (up to 80%) in newborns than in adults, with diminished biological activity [19]. Levels increase after birth, with some complement factors reaching adult concentrations within a month but others evolving much more slowly [19].

An adaptive immune response does occur in newborns, but it is slower and skewed towards T helper-2 (Th-2) reactions against extracellular pathogens [24]. After birth, innate lymphoid cells, which are critical regulators of innate immunity and inflammation at barrier surfaces (e.g., skin, respiratory and gastrointestinal tracts), indirectly modulate adaptive immunity via interactions with stromal cells in lymphoid tissues and epithelial cells at barrier surfaces [26]. Contact with the hostile environment drives cells of the innate and the adaptive mucosal and systemic immune systems to mature and expand, and the immunologic competence of the baby expands rapidly over the first few months of life [11]. Defenses against intracellular pathogens and cell-mediated immunity rely on Th-1 responses, which reach adult levels only after around two years of age [24]. Microbial antigens are essential for the education of the immune system and development of Th-1 type responses and breakdown in such immune education may predispose to allergic, inflammatory and autoimmune diseases [5,10].

As children grow and develop, their immune systems continue to mature and acquire memory after exposure to multiple foreign challenges including from pathogens, food and other environmental components and vaccines [19]. Neutrophil concentrations are increased in children aged 1–6 years compared with infants (but are still only half the adult levels), as are eosinophil and basophil concentrations (both then decrease with age); lymphocyte and platelet counts are lower in children compared with infants and steadily decline with age [25]. Closer analysis of lymphocyte subtypes indicates that the proportion of different lymphocyte subsets changes over time [25]. For example, the percentage of CD3^+^ T cells (required for activation of CD4^+^ and CD8^+^ T cells) is significantly higher in children than in infants. However, the proportion of CD4^+^ T cells is significantly lower in children than in infants [25]. CD4^+^ helper T cells recognize peptides presented by major histocompatibility complex (MHC) II molecules found on antigen-presenting cells, and subsequently secrete cytokines that facilitate different immune responses according to the source of the antigen [27]. In contrast, the percentage of CD8^+^ T cells is significantly higher in children than in infants and steadily increases over time [25]. CD8^+^ cytotoxic T cells recognize peptides presented by MHC I molecules found on all nucleated cells, and secrete cytokines like tumor-necrosis factor alpha or interferon gamma to help to kill infected or malignant cells [27]. Analysis of B cells indicates that the proportion of CD19^+^ cells is highest in infants and children and decreases significantly thereafter [25]. CD19 is an antigen that is present on all B cells, is involved in signaling, and is a biomarker for B lymphocyte development [28]. Antibody production increases with age from infancy to childhood. For example, adult levels of IgG (expressed on the surface of mature B cells, and the most prevalent immunoglobulin in serum) are reached by the age of 11–12 years, with a further increase during puberty, while levels of IgA (the second most prevalent immunoglobulin in serum, which can activate the complement pathway) continue to increase past puberty until they reach adult levels; in contrast, adult levels of IgM (the first immunoglobulin made by the fetus and virgin B cells challenged with antigen) are reached by the age of four years [29].

### 2.2. Adolescents and Adults

After childhood, physical changes occur in lymphoid tissues, which support immune responses and are responsible for producing lymphocytes and antibodies. For example, thymic tissue in the thymus (the organ that is instrumental in the production and maturation of T cells before birth and throughout childhood) is gradually replaced by adipose tissue after puberty and gives the impression of being larger in children and becoming smaller after adolescence [27]. The functional portion of the gland is considerably reduced (known as involution), but the thymus populates secondary lymphatic organs and tissues with T cells [27]. T cells continue to be produced in the thymus throughout a person’s lifetime, although to a much smaller extent [27], but it is thought that adults rely on the naïve T cell pool produced mostly before puberty [30]. There is a progressive decline in the percentage of total lymphocytes and absolute numbers of T and B cells in the blood from infancy to adulthood [25]. However, there is a significant increase in all T cell subsets (CD3^+^, CD4^+^, and CD8^+^) in adults compared with children, and a decrease in the biomarker for B lymphocyte development, CD19 [25]. There is also a significant increase in the number of NK cells in adolescents compared with infants and children, as well as in adults compared with infants (but not children) [25].

It should be noted that the immune system reaches maturity by adulthood, and small decreases or increases in single selected markers of immune function may not be clinically important after that. In general, young, non-pregnant adults seem to be well equipped to cope with immune challenges, which may reflect the procreative potential of young adults in the survival of the species [19]. However, there are some sex-specific differences that are evident in the prevalence of certain diseases. For example, autoimmune disorders such as Sjogren syndrome, systemic lupus erythematosus and autoimmune thyroid disease are higher in women [23]. The inflammatory immune response differs between men and women, with females generating higher proinflammatory cytokine and chemokine responses to the influenza virus and experiencing greater morbidity and mortality than males [31]. Women also initiate a higher humoral immune response to the influenza vaccine, and experience more adverse reactions than men [31]. However, the raised immunity in females following vaccination leads to greater cross-protection against novel influenza viruses compared with men [31]. It is thought that women typically mount stronger immune responses than men because of the immunomodulatory effects of estrogen in women and the humoral immunity suppressing effects of testosterone in men; however, the full extent of sex on functional immune responses remains unclear [23].

### 2.3. Older People

As the body ages, so does the immune system [32] and most older people over the age of 60–65 years (although not all) experience some immune dysregulation that makes them less able to respond to immune challenges [33,34]. There is a loss of lymphoid tissue, particularly in the thymus, with increasing age [25], and the ability to respond to pathogens, antigens and mitogens decreases [5,33]. The development of long-term immune memory is also impaired, with a diminished response to vaccination [5]. This is commonly referred to as immunosenescence, which mostly seems to affect adaptive immunity but also the innate immune system to a lesser extent [32].

Immune cells are constantly renewed from hematopoietic stem cells but these mature with age and become less able to produce lymphocytes; furthermore, the total amount of hematopoietic tissue decreases [34,35]. A loss of immune cells and a decrease in the number of circulating lymphocytes are characteristic in the immune systems of older people [23], consistent with reduced production of T cells in the involuted thymus, as well as diminished function of mature lymphocytes in secondary lymphoid tissues [34,36]. The proportions of naïve T cell subsets also change with age; for example, CD3^+^ and CD8^+^ cytotoxic T cells decrease significantly in older people, but CD4^+^ helper T cells increase from adolescence to adulthood and then stabilize in older people [25], suggesting that CD4^+^ cells are subject to stricter homeostatic mechanisms given their importance in immune system function [1]. On the other hand, memory T cells accumulate, especially late-stage differentiated CD8^+^ cells [30]. CD19^+^ cells decrease significantly from childhood to old age [25]. The total number of naïve B cells remains unchanged with ageing; instead, there is a decrease in memory B cells that may occur secondary to T cell deficiencies [34]. The incidence of autoimmune diseases also increases in later life, as the ageing immune system becomes unable to fully tolerate self-antigens [19,37]. Age-related lymphopenia may lead to a decrease in regulatory T cell function, an increase in T cells with increased affinity to self- or neoantigens, an increased prevalence of autoantibodies, and decreased clearance of apoptotic cells by macrophages [19,33].

Changes in the innate immune system also occur with increasing age. Skin and mucous membranes—the first line of defense against invading pathogens—become less effective as skin cell replacement declines and dermal and subcutaneous atrophy occurs [1]. After 60 years of age, there is a decrease in secretory IgA, which forms part of the first line of defense against pathogens that manage to invade the mucosal surfaces [38]. In older people, functional activity of immune cells such as phagocytes and the intracellular respiratory burst necessary to kill pathogens are reduced [1]. Although healthy ageing does not seem to affect the overall number of dendritic cells, which are responsible for the recognition and phagocytosis of pathogens, processing of antigens, priming of naïve T cells and regulation of the response of B and NK cells [1], they are diminished in certain areas such as Langerhans cells in the skin [39]. However, their ability to recognize invading pathogens is impaired by compromised Toll-like receptors on dendritic cells, for example, which is known to occur in ageing [40]. This reduces their ability to induce proinflammatory cytokine production and regulate antigen presentation to naïve T cells, and to activate antigen-specific adaptive immune responses [41]. The number of NK cells increases significantly in older people compared with younger adults [1,25], which may be the result of an accumulation of long-lived NK cells [42]. However, there is not an accompanying increase in cytotoxicity, but instead a decrease in the functioning of the NK cells, including a slower resolution of inflammatory responses [43].

In fact, a longer inflammatory process is induced in older adults [3]. Increased levels of circulating pro-inflammatory cytokines (e.g., tumor-necrosis factor alpha, IL-1, and IL-6 [1,25]) characterize low-grade chronic inflammation in older people, a process known as inflamm-aging [1]. Inflamm-aging is a physiological response to lifelong antigenic stress and, if kept under control by anti-inflammatory cytokines such as IL-10 [1] represents an efficient defense mechanism in older people. Increased production of anti-inflammatory molecules is an essential counter-regulatory process in ageing, as inflamm-aging would otherwise be damaging [44]. Many of the most common chronic diseases associated with ageing, such as atherosclerosis, Alzheimer’s disease, osteoporosis and diabetes [1], are related to low-grade inflammation [32]. Oxidative stress also has a role in inflamm-aging, emphasizing the role of oxidative stress in the complex mechanisms of ageing [44]. Immune cells, which contain a high percentage of polyunsaturated fatty acids in their plasma membrane and so are susceptible to lipid peroxidation, are particularly sensitive to changes in the oxidant–antioxidant balance [10]. Thus, oxidative damage can compromise the integrity of immune cell membranes and alter transmission of signals both within and between different immune cells, leading to an impaired immune response [10]. It has been suggested that, in older people, many immune markers of immunosenescence may actually be more related to prolonged exposure to antigen stimulation and to oxidative stress involving the production of reactive oxygen species (ROS), rather than to “ageing” of the immune system per se [23,35,36]. For example, in modern industrialized populations, the cumulative effect of antigenic exposure may be lower than in less hygienic societies [30]. One individual may experience different environmental factors at different stages of life compared to another, and thus their immune profiles will also differ [23]. Some older people age without any major health problems, known as healthy ageing, and immune system dysfunction appears to be mitigated in this population [1]. Genetic and environmental factors (e.g., good nutritional status) may play a role, but these have yet to be described. It may be that the only truly universal age-related changes in immune markers are the reduction in the numbers and proportions of peripheral blood naïve T cells, due mainly to thymic involution, reflecting the aging of the hematopoietic stem cell system [36].

## 3. Response to Infection

The nature of the response of the immune system to a pathogen is initially dependent on whether the innate immune defenses can eliminate the infectious organism. If not, previous experience with the pathogen will determine how rapidly T and B cells in the adaptive immune system are able to mount a defense against it, supported by the innate immune system. Certain factors may affect the response of the immune system to infection.

### 3.1. Infants and Children

The developing immune system is still functionally immature in infants and young children. The innate immune system is relatively susceptible to pathogens, while the adaptive immune system is less able to quickly respond to T-cell-dependent antigens, especially in babies [19]. These factors, combined with their greater potential for exposure to pathogens at nursery and school, means that infants and young children are more susceptible to infections than adolescents and adults [23]. Vaccinations have been developed to combat common but potentially deadly infections (e.g., meningococcal bacteria, diphtheria, polio, pertussis, etc.), administered from around eight weeks after birth (when passive immunity begins to wane) and throughout childhood.

Although most childhood infections happen only once (e.g., chickenpox, measles, and mumps), followed by lifelong protection [19], many rhinoviruses can cause the common cold and reinfection is common. For example, children less than one year old have been noted to experience an average of six colds per year; the frequency decreases with age to about three colds per year in older children (10–14 years) [45]. Males are more often affected than females before three years of age, while the reverse is true in older children [45]. Infection with the seasonal influenza virus, which is caused by a different influenza type each year, is also more common in children under the age of five years [46]. In this age group, symptoms of flu can cause severe illness, complications and even death [46]. Sickness and diarrhea frequently occur in childhood, with many children in industrialized countries experiencing more than one episode of infective gastroenteritis per year, usually caused by rotavirus [47]. The frequency is exacerbated by close contact with other children and often less-than-optimal hygienic practices [47]. Lower respiratory tract infections (e.g., bronchitis and pneumonia) are more common in children under five years old than any other age group worldwide, and risk factors include air pollution and suboptimal breastfeeding [48]. Micronutrient deficiencies also have immunological consequences in infants and young children, and can increase morbidity and mortality from many diseases, including pneumonia, diarrheal disease, and measles [4,49]. Infection and undernutrition have a synergistic relationship, and micronutrient deficiencies cause specific immune impairments that affect both the innate and adaptive immune systems, such as impaired phagocyte and lymphocyte activity with zinc deficiency, or compromised development of neutrophils, macrophages and NK cells with vitamin A deficiency [50].

### 3.2. Adolescents and Adults

**Figure 2:**
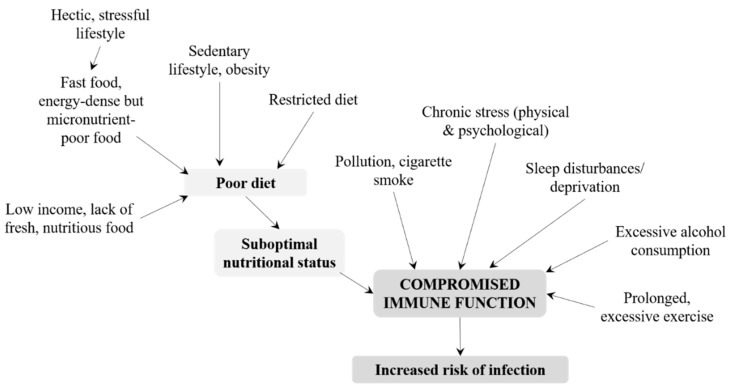
Life-style factors affecting immune function during adulthood. The risk of infection is also influenced by gender, early programming, vaccination history, pathogen exposure, specific health conditions, and diseases.

Immunological maturity is achieved by adolescence, and young adults should be well fortified against attack by pathogens [19]. Nevertheless, several lifestyle-related factors affect immune competence in healthy adults and increase their risk of infection (Figure 2). In particular, nutritional status can be compromised by a poor diet, which is often observed in adults with a hectic and stressful lifestyle and ready access to fast food or energy-dense, micronutrient-poor convenience food. Essential micronutrients such as vitamin B12 may be lacking in vegetarians and vegans, while adults in low-income families may be unable to afford fresh, nutritious foods. As outlined in Table 1, micronutrients have essential roles in the immune system and an inadequate intake may have deleterious effects [4]. A poor diet may be combined with a sedentary lifestyle, leading to obesity, suboptimal immune response, and increased risk of infection [51]. However, prolonged and excessive exercise and overtraining are also thought to impair immune function [52,53,54]. However, this view has recently been disputed; instead, it is suggested that regular physical activities might be beneficial for immunological health and limit or delay age-associated changes to the cellular composition of the adaptive immune system (for example, by countering the expansion of memory T cells that may contribute to systemic inflammation) [55]. Nevertheless, prolonged bouts of exercise and heavy training regimens in adults may create an imbalance between ROS and antioxidant defenses [54], leading to oxidative stress that alters signal transmission in the immune system and impairs the immune response [10]. Pollution and cigarette smoke certainly compromise immune function, particularly when combined with poor nutrition [10]. Reactive oxygen species in, and caused by, pollution can also upset the oxidant–antioxidant balance within the body and cause oxidative stress, which must be counteracted by an adequate supply of antioxidants [10]. Chronic, psychological stress is another factor that can impact immune function, suppressing cellular and humoral responses [56]. Alcohol consumption has variable effects on immunity; moderate amounts of polyphenol-rich alcoholic beverages potentially provide some immune protection while excessive consumption of alcohol can suppress many aspects of immune function and consequently increase the risk of infection [57]. Sleep is an important homeostatic regulator of immune function and plays a specific role in immunological memory [58]. Sleep disturbances and deprivation are therefore likely to have adverse effects on the immune system, including dysregulation of NK cells and pro-inflammatory and anti-inflammatory cytokines [58].

These factors, alone or in combination, weaken the immune system in adults and can increase the risk of infection. The incidence of common cold is lowest in adolescents compared with all other age groups, but increases in adults aged 20–30 years [45]; the risk is likely to be greater in those who come into close contact with children, who are at highest risk. Common cold is also more likely in those suffering from psychological stress [59], while moderate physical exercise may decrease the risk [60]. Infection with influenza viruses other than the seasonal variety (e.g., H1N1) is more prevalent in young to middle-aged, previously healthy adults [61]. In contrast to children, sickness and diarrhea in adults are often caused by norovirus [62] and campylobacter [63]. Worldwide, norovirus causes 685 million cases of acute gastroenteritis every year in adults [64].

### 3.3. Older People

In older people, a lifetime of exposure to antigens and to numerous sources of oxidative stress can cause immune dysregulation that makes them more susceptible to infections than any other age group apart from young children [23,35,36]. Immune memory can be very long lasting, providing protection against many infections for decades; however, people are living much longer than before, and the pool of antigen-specific T cells may diminish over time [36]. In addition, thymic involution and the relative paucity of naïve lymphocytes in older people means that they are less able to mount an adequate defense against neoantigens and thus exposure to them is more hazardous than in younger people [36].

Although certain infections are less likely in older people (for example, the incidence of common cold has been shown to be the lowest those aged over 60 years [45]), the risk of many others such as urinary tract infections, lower respiratory tract infections, skin and soft tissue infections, for example, is greatly increased [65]. Furthermore, this age group is more likely to suffer prolonged infections, severe symptoms and secondary complications [33]. Around two-thirds of older patients with common cold develop lower respiratory illness [66], while older individuals are 2–10 times more likely to die of infection than younger people [11]. In those aged 70 years or older, 1.27 million deaths were thought to be caused by lower respiratory tract infections in 2015 [48]. Infection with seasonal influenza viruses is normally greatest in older people and young children [46]. Although influenza is not a life-threatening illness in most adults [67], in industrialized countries, influenza-associated deaths occur most often among people aged 65 years or older [46]. The greater morbidity and mortality associated with influenza in this age group occur because dysregulation in the immune response predisposes them to secondary bacterial infection of the respiratory tract (e.g., bronchitis and bacterial pneumonia) [68]. Protection against infection is dependent on T-cell-mediated responses and any dysregulation can impair the ability to mount a T-cell response, especially if there is also infection with cytomegalovirus [36]. This is the case in many older people, and these factors may explain why they have a poorer response to vaccines than the young [1,36]. Nevertheless, influenza vaccination can reduce severe illnesses and complications in people aged 65 years or older [46].

## 4. Micronutrient Requirements and Reported Deficiencies

The development, maintenance and optional functioning of immune cells is dependent on adequate nutrition, evident at all stages of life [4,5,33,49,69]. Key immunomodulatory roles of certain micronutrients are outlined in Table 1. Immune defenses can be impaired by undernutrition, which increases susceptibility to infection [4,5,70]. In turn, infection can cause a significant increase in the demand for micronutrients, met by endogenous or exogenous (i.e., the diet) supplies [5,50]. Vitamins A (and beta carotene), C, D, B2, and B12, folic acid, iron, zinc and selenium are just some micronutrients that have immunomodulatory and/or antioxidant effects and thus influence the susceptibility of a host to infectious diseases, as well as the course and outcome of infection [70].

### 4.1. Infants and Children

In babies and infants, breast milk is the major nutritional influence and is formulated to ensure that nutritional needs are met [49,71]. Breastmilk contains various immunological components such as antibodies (e.g., antigen-specific IgA), anti-inflammatory cytokines and other antimicrobial factors, but also most of the micronutrients necessary to support neonatal development, including of the immune system [49,71]. The concentrations of certain micronutrients in breastmilk (e.g., calcium, magnesium, and copper) are regulated by maternal homeostatic mechanisms (i.e., independent of maternal nutritional status and diet) to ensure they are sufficient to meet infant needs [72] and to protect them against deficiency or excess [71,73,74,75]. However, human milk is a poor source of iron and zinc and the needs of the child cannot be met by breast milk alone for zinc, or beyond six months for iron [72]. In contrast, the excretion of fat- and water-soluble micronutrients (e.g., vitamin A, and vitamins B1, B2, B6, B12, and C, respectively) into breast milk is dependent on maternal intake and varies worldwide [71,74,75]. Furthermore, vitamin D content of human milk is low and usually insufficient to meet requirements in exclusively-breastfed infants if the infant’s sunlight exposure is limited [72,76]. During weaning and in the first years of life, both vitamin A and zinc play major roles in immunity to infectious disease [77].

Children do not need micronutrients in the same intakes as adults [78] (Table 2), and lower amounts are adequate to fulfill their various roles throughout the body, including within the immune system. Nevertheless, micronutrient deficiencies are prevalent in infants and preschool children in developing and low- to middle-income countries (e.g., [17,79,80,81,82]), and this age group is at the highest risk of multiple micronutrient deficiencies [83]. Worldwide, the three most common deficiencies are for iron, vitamin A and iodine [84], but zinc deficiencies are also common [83]. In young children, mainly in industrialized countries, deficiencies may occur because many micronutrients (e.g., vitamin C and B vitamins) are found in fruit and green, leafy vegetables and children are often fussy about what they eat. However, there are few data on micronutrient levels in infants from high-income countries. The available data suggest that, even in industrialized countries, some infants who are breastfed may not be receiving optimal amounts of certain micronutrients, as the levels found in breastmilk, maternal serum or urine did not always reach recommended levels in all women [73,85,86,87,88,89,90,91,92,93,94,95]. Reported micronutrient deficiencies in Europe [96] compared with recommended dietary allowances (RDA) [78] are shown in Table 2. It can be seen from the upper values that some children between the ages of 4 and 14 years had a surfeit of many micronutrients included in the table. However, the lower ranges indicate that there were children who had an insufficient intake of vitamin D (all ages), vitamin A (females 10+ years), vitamin E, folate, zinc (10+ years), iron (all ages) and selenium (all ages). Only the intakes of vitamins C, B6 and B12 and copper were sufficient within this age range.

### 4.2. Adolescents and Adults

An adequate amount of all micronutrients is required for optimal immune function in adolescents and adults (and throughout life), but in higher amounts compared with infants and children [78] (Table 2). It is especially important to ensure that antioxidant levels (e.g., vitamins C, E, and A) and micronutrients that are components of antioxidant enzymes (e.g., zinc, copper, iron, and selenium) are sufficient to combat the oxidative stress that is induced by many lifestyle factors common in this group, and which has great impact on immune function [10,23,35,36,44]. An adequate supply of micronutrients that affect the thymus is also important; for example, even marginal zinc deficiency is known to result in thymic atrophy and can increase the risk of infection [97]. Vitamin D intake is usually inadequate in most age groups worldwide, even in countries with mandatory food fortification [98], which can increase the risk of infection, especially respiratory tract infections [71].

Micronutrient deficiencies have been recorded in adolescents and adults in Europe [96] (Table 2). The lower ranges indicate that some adolescents had an insufficient intake of vitamin C (males 15–18 years), vitamin D, vitamin A (males 15–18 years; females 10–18 years), vitamin E, folate, zinc (10–18 years), iron and selenium. Only the intakes of vitamins B6 and B12 and copper were sufficient in all cases. In adults, there were insufficient dietary intakes for all micronutrients shown, apart from vitamin B6 and copper. Intakes were particularly low in female adults for folate, iron and selenium.

### 4.3. Older People

Although the recommended dietary allowances for older people indicate that their energy needs are lower than their younger counterparts, micronutrient requirements are mostly the same [78] (Table 2). However, micronutrient deficiencies are common in older people; it has been estimated that 35% of those aged 50 years or older in Europe, USA and Canada have a demonstrable deficiency of one or more micronutrients [33]. Many older people have chronic health conditions requiring hospitalization, live in care homes, or tend to eat less and make different food choices (e.g., choosing low nutrient density, often cheaper, and foods) [99,100]. An insufficient intake of micronutrients in older people has been reported both in the community (vitamins A, B12, D and zinc) and at a higher prevalence in long-term care facilities (vitamins A, D, and E) [101], while lower food intake has been associated with lower intakes of calcium, iron, zinc, B vitamins and vitamin E in older people [100]. Overall, data from Europe [96] (Table 2) suggest that there is an insufficient intake of most micronutrients in older people, apart from vitamin B12, iron and copper [96]. In particular, intakes were low for vitamin D (females), vitamin E (males and females) and folate (males and females). Older women, who usually have a longer life expectancy compared to men, are often at higher risk of deficiency, especially for vitamins B12, A, C, and D, iron and zinc [99]. Furthermore, menopause affects utilization of micronutrients; for example, vitamin C gradually decreases as menopause advances, correlated negatively to body mass index [102]. As in younger adults, a sufficient supply of antioxidants (e.g., vitamin C, selenium, and zinc) is required to combat the oxidative stress that is a major factor in immune dysregulation in older people. However, older people lose their ability to produce endogenous antioxidants compared with younger adults [103]. The skin of older adults is less able to synthesize vitamin D, and synthesis is about 75% slower in people aged 65 years than in younger adults [17].

## 5. Clinical Impact of Micronutrient Deficiencies and Supplementation

An inadequate intake of micronutrients at any stage of life affects various functions within the immune system, manifesting in decreased resistance to infections and an increase in the severity of symptoms (Table 3). For example, zinc deficiency can increase thymic atrophy, decrease lymphocyte number and activity, and increase oxidative stress and inflammation by altering cytokine production [14,97]. As a result, the risk of all types of infection (bacterial, viral, and fungal), but especially diarrhea and pneumonia, is increased [49]. A low vitamin C status also increases susceptibility to infections such as pneumonia [71], possibly because low levels of antioxidants such as vitamin C are unable to counteract the oxidative stress observed in pneumonia [104]. Increased production of ROS during the immune response to pathogens may decrease vitamin C levels further [105]. Vitamin D deficiency increases the risk of infection and autoimmune diseases such as multiple sclerosis and diabetes, probably related to activity of vitamin D receptors, which are found throughout the immune system [106,107].

Considering the importance of micronutrients in immunity, and the fact that many people of all ages have single or multiple micronutrient deficiencies that can have detrimental immunological effects, there is a rationale for micronutrient supplementation to restore concentrations to recommended levels, especially after an infection, and to support immune function and maintenance. To avoid any unwanted side effects, it is of course important to ensure that supplementation does not exceed recommended tolerable upper intake levels (Table 2), the highest level of daily nutrient intake that is likely to pose no risk of adverse health effects in most people [78]. Although this is theoretically possible, the reported micronutrient intake data in Table 2 suggest that over-supplementation is unlikely with most micronutrients, perhaps with the exception of vitamin A in children. It should be noted that the safety margins in micronutrient supplements ensure that proper consumption does not result in over-supplementation, and that food supplement labels should be carefully read to avoid misuse and the potential for over-supplementation.

As no single biomarker exists that accurately reflects the effects of supplementation on the immune response, clinical outcomes are instead used to determine the effectiveness of supplementation [49,69].

### 5.1. Infants and Children

Micronutrient deficiencies are closely linked to infectious diseases that can cause substantial morbidity and mortality in infants and children [49]. Worldwide, micronutrient supplementation studies have looked at the effects of vitamins D, A and E and minerals such as iron, selenium and zinc [49]. Zinc supplementation reduces morbidity and mortality from infectious diseases among infants and children in developing countries [77]. In low-birthweight infants, supplementary zinc can partly restore cell-mediated immunity [33]. Zinc can also reduce both the risk and duration of pneumonia in children, help to manage infantile diarrhea, lead to fewer episodes of malaria, and reduce the duration of diarrhea [3,17,71]. The duration and severity of common cold symptoms can be reduced by zinc supplementation in children when taken within 24 h of symptom onset [108]. Similar results have been observed with vitamin C, which shortened the duration of a cold in children (especially with higher doses) and reduced the severity of symptoms; a greater effect was observed in children compared with adults, including a greater prophylactic effect of vitamin C [105]. Both zinc and vitamin C may also improve the outcome of pneumonia, malaria and diarrheal infections in children [9]. In children with vitamin A deficiency, supplementation can decrease the risk of morbidity and mortality from infectious diseases [77], and reduce the incidence of diarrhea and measles [14,71].

### 5.2. Adolescents and Adults

Supplementation with vitamin C reduces the duration and severity of common cold symptoms in adults [105]. In those under physical stress (e.g., at work, during sports, and under extreme temperatures) [104], or in cases where vitamin C levels are slightly below recommended levels, vitamin C supplementation reduces common cold incidence. For example, in young males with marginal vitamin C deficiency, supplementation was shown to reduce the incidence of common cold and the duration of cold symptoms compared with placebo, accompanied by improved activity levels [109]. When used in combination with zinc, vitamin C supplementation can relieve symptoms such as rhinorrhea in common cold [110], which is commonly regarded as the most frequent and troublesome symptoms of the infection (along with nasal congestion) [111]. Supplementation with vitamin D can protect against respiratory tract infections and reduce the risk of acute respiratory illness and influenza, especially with once-daily dosing [112,113,114,115]. Benefits are particularly apparent in those who are very vitamin D deficient [115]. In light of their positive effects on respiratory tract infections, it has been suggested that there is a good rationale to combine vitamins C and D with zinc to support immune functions and help minimize the risk of infection [3]. Supplementation with multiple micronutrients has beneficial effects on the symptoms associated with the so-called “sick building syndrome”, associated with prolonged contact with environmental factors that act as vehicles for pollutants [10]. Significantly fewer adults who received the micronutrient supplement reported headache, sore eyes, nasal congestion, throat inflammation, tiredness/pain, diarrhea or symptoms associated with an acute respiratory tract infection, such as cough [10].

### 5.3. Older People

Impaired immunity in older people, often caused by multiple micronutrient deficiencies, is evident in the increased incidence and severity of common infections that affect the upper and lower respiratory tracts, as well as the urinary and genital tracts [33,116]. Supplementation with modest amounts of a combination of micronutrients can have beneficial effects [33]. Higher levels of CD4^+^ and CD8^+^ T cells and an increased lymphocyte proliferative response to mitogens have been observed with vitamin A, C and E supplementation [117], while micronutrient supplementation with higher levels of vitamins C, E and beta-carotene increased the number of various subsets of T-cells, enhanced lymphocyte response to mitogen, increased IL-2 production and NK-cell activity, increased the response to the influenza virus vaccine, and led to fewer days of infection [118]. Supplementation with a complex micronutrient formulation in older people increased the number of various types of immune cells, including total lymphocytes, and induced a shift from memory T cells to naïve T cells [119]. Multiple micronutrient supplementation in older people may also reduce antibiotic usage and lead to higher post-vaccination immune responses [33].

Marginal zinc deficiency is common in older people, as their dietary intakes are generally lower and plasma zinc concentrations decline with age, possibly connected to impaired absorption, alterations in cellular uptake, and epigenetic dysregulation of DNA methylation or the methionine/transsulfuration pathway, for example [14]. Supplementation with low to moderate doses of zinc in healthy older people can help to restore thymulin activity, increase the numbers of cytotoxic T cells, reduce the number of activated Th cells (which contribute to autoimmunity) and increase the cytotoxicity of NK cells [14], immunological benefits that help to reduce the incidence of infections such as common cold, cold sores and influenza [120], as well as the incidence and morbidity of pneumonia [121]. There are some reports that an adequate zinc supply could prevent degenerative age-related diseases including infection and cancer [122]. Sufficient vitamin C is also important in older people, who are at risk of vitamin C deficiency, especially females [96]. Adequate vitamin C intakes can optimize cell and tissue levels and help to protect against respiratory and systemic infections (e.g., reduced duration and severity of pneumonia [71]), while higher levels are required during infection to compensate for the increased inflammatory response and metabolic demand induced by the pathogen, and thus help to reduce the duration and severity of symptoms [12]. Supplementation with vitamin E in older people has been shown to significantly improve NK cytotoxic activity, neutrophil chemotaxis and the phagocytic response, and enhance mitogen-induced lymphocyte proliferation and IL-2 production [123]. Vitamin E can also improve T-cell-mediated immunity and increase the production of antibodies in response to the hepatitis B and tetanus vaccines [124]. The risk of upper respiratory tract infections, especially common cold, was significantly lower after vitamin E supplementation in nursing home residents, although there was no apparent effect on lower respiratory tract infections [125]. However, not all studies have reported beneficial effects on respiratory tract infections with vitamin E supplementation in older people [14].

## 6. Conclusions

**Figure 3:**
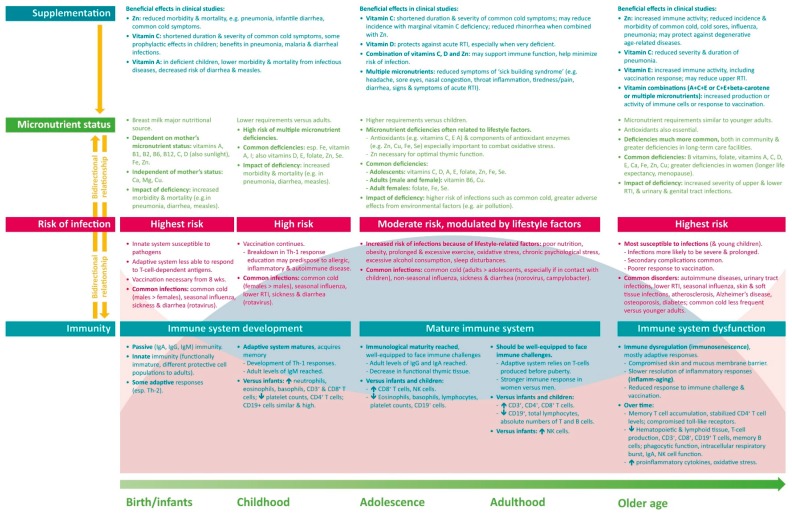
Differences in immunity and nutrition over a lifetime. Ca, calcium; Cu, copper; Fe, iron; I, iodine; Ig, immunoglobulin; Mg, magnesium; NK, natural killer; RTI, respiratory tract infections; Se, selenium; Th, T helper cell; Zn, zinc.

The immune system undergoes many changes over the life course—developing and maturing during childhood, potentially achieving peak function in early adulthood, and gradually declining in most people in older age (Figure 3). Distinct immune features are present during each life stage, and specific factors differentially affect immune function, with a resulting difference in the type, prevalence and severity of infections with age. A common factor throughout life is the need for an adequate supply of micronutrients, which play key roles in supporting immune function. Multiple micronutrient deficiencies are common throughout the world, with the likelihood increasing with age. Tailored supplementation based on the specific needs of each age group may help to provide an adequate basis for optimal immune function. The available clinical data suggest that micronutrient supplementation can reduce the risk and severity of infection and support a faster recovery. However, much more research is required into the effects of micronutrient supplementation on immune functions and on clinical outcomes. Nevertheless, current knowledge regarding the importance of micronutrients in immunity, the effects of micronutrient deficiencies on the risk and severity of infection, and the worldwide prevalence of an inadequate micronutrient status form a sound basis for the use of a targeted multiple micronutrient supplement to support immunity over a person’s lifetime.

## Figures and Tables

**Table 1:** Overview of key roles played by select micronutrients in the immune system [4,9,10,11,12,13,14].

Micronutrient/Role	Innate Immunity	Adaptive Immunity
Vitamin C	Effective antioxidant that protects against ROS and RNS produced when pathogens are killed by immune cells [9,14] Regenerates other important antioxidants such as glutathione and vitamin E to their active state [9] Promotes collagen synthesis, thereby supporting the integrity of epithelial barriers [10] Stimulates production, function and movement of leukocytes (e.g., neutrophils, lymphocytes, phagocytes) [9,14] Increases serum levels of complement proteins [14] Has roles in antimicrobial and NK cell activities and chemotaxis [10] Involved in apoptosis and clearance of spent neutrophils from sites of infection by macrophages [12]	Can increase serum levels of antibodies [12,14] Has roles in lymphocyte differentiation and proliferation [10,12]
Vitamin D	Vitamin D receptor expressed in innate immune cells (e.g., monocytes, macrophages, dendritic cells) [14] Increases the differentiation of monocytes to macrophages [10] Stimulates immune cell proliferation and cytokine production and helps protect against infection caused by pathogens [14] 1,25-dihydroxyvitamin D_3_, the active form of vitamin D, regulates the antimicrobial proteins cathelicidin and defensin, which can directly kill pathogens, especially bacteria [14]	Mainly inhibitory effect in adaptive immunity [14]; for example, 1,25-dihydroxyvitamin D_3_ suppresses antibody production by B cells and inhibits T cell proliferation [14]
Vitamin A	Helps maintain structural and functional integrity of mucosal cells in innate barriers (e.g., skin, respiratory tract, etc.) [14] Important for normal functioning of innate immune cells (e.g., NK cells, macrophages, neutrophils) [14]	Necessary for proper functioning of T and B lymphocytes, and thus for generation of antibody responses to antigen [14] Involved in development and differentiation of Th1 and Th2 cells and supports Th2 anti-inflammatory response [10]
Vitamin E	An important fat-soluble antioxidant [10] Protects the integrity of cell membranes from damage caused by free radicals [14] Enhances IL-2 production and NK cell cytotoxic activity [10]	Enhances T cell-mediated functions and lymphocyte proliferation [10] Optimizes and enhances Th1 and suppresses Th2 response [10]
Vitamin B6	Helps regulate inflammation [13] Has roles in cytokine production and NK cell activity [13,15]	Required in the endogenous synthesis and metabolism of amino acids, the building blocks of cytokines and antibodies [14] Has roles in lymphocyte proliferation, differentiation and maturation [14] Maintains Th1 immune response [10] Has roles in antibody production [13]
Vitamin B12	Has roles in NK cell functions [13]	May act as an immunomodulator for cellular immunity, especially with effects on cytotoxic cells (NK cells, CD8^+^ T-cells) [10] Facilitates production of T lymphocytes [13] Involved in humoral and cellular immunity and one-carbon metabolism (interactions with folate) [13]
Folate	Maintains innate immunity (NK cells) [10]	Has roles in cell-mediated immunity [13] Important for sufficient antibody response to antigens [13] Supports Th 1-mediated immune response [13]
Zinc	Antioxidant effects protect against ROS and RNS [9] Helps modulate cytokine release and induces proliferation of CD8^+^ T cells [10,16] Helps maintain skin and mucosal membrane integrity [10]	Central role in cellular growth and differentiation of immune cells that have a rapid differentiation and turnover [17] Essential for intracellular binding of tyrosine kinase to T cell receptors, required for T lymphocyte development and activation [9] Supports Th1 response [10]
Iron	Involved in regulation of cytokine production and action [10] Forms highly-toxic hydroxyl radicals, thus involved in the process of killing bacteria by neutrophils [10] Important in the generation of ROS that kill pathogens [14]	Important in the differentiation and proliferation of T lymphocytes [14] Essential for cell differentiation and growth, component of enzymes critical for functioning of immune cells (e.g., ribonucleotide reductase involved in DNA synthesis) [10]
Copper	Free-radical scavenger [4] Antimicrobial properties [14] Accumulates at sites of inflammation, important for IL-2 production and response [13,14] May play a role in the innate immune response to bacterial infections [14]	Has roles in T cell proliferation [13] Has roles in antibody production and cellular immunity [18]
Selenium	Essential for the function of selenium-dependent enzymes (selenoproteins) that can act as redox regulators and cellular antioxidants, potentially counteracting ROS [10,14] Selenoproteins are important for the antioxidant host defense system affecting leukocyte and NK cell function [13]	Involved in T lymphocyte proliferation [4,13] Has roles in the humoral system (e.g., immunoglobulin production) [13]

IL, interleukin; NK, natural killer; RNS, reactive nitrogen species; ROS, reactive oxygen species; Th, helper T cell.

**Table 2:** Life-stage-specific micronutrient deficiencies in Europe. Reported micronutrient intakes that are below the recommended dietary allowance are shown in bold. The table also shows the tolerable upper intake levels, the highest level of daily nutrient intake that is likely to pose no risk of adverse health effects in most people.

**Select Micronutrients**	**Recommended Dietary Allowance** [78]			**Tolerable Upper Intake Levels** [78]			**Reported Mean Micronutrient Intakes, Min–Max** [96]		
	**Children ^a^** 4–8 years 9–13 years 14–18 years: M/F	**Adults** 19–50 years: M/F ^b^	**Older age** 51 to >70 years: M/F	**Children ^a^** 4–8 years 9–13 years 14–18 years	**Adults** 19–50 years: ^b^	**Older age** 51 to >70 years	**Children** 4–6 years: M/F 7–9 years: M/F 10–14 years: M/F 15–18 years: M/F	**Adults** 19–50 years: M/F	**Older age** 51 to >70 years: M/F
Vitamin C, mg/day	25 45 75/65	90/75	90/75	650 1200 1800/1800	2000	2000	60–157/61–157 63–172/57–172 73–197/77–222 71–201/67–205	64–153/62–153	59–142/60–160
Vitamin D, μg/day	15	15	15–20	75 100 100/100	100	100	1.8–5.8/1.5–6.5 1.5–6.4/1.5–5.1 1.5–4.8/1.2–4.5 1.8–7.5/1.5–7.1	1.6–10.9/1.2–10.1	0.7–15.0/0.7–12.9
Vitamin A, μg/day	400 600 900/700	900/700	900/700	900 1700 2800/2800	3000	3000	400–1100/400–1200 400–1300/400–1100 400–2400/300–2300 400–1800/300–1600	500–2200/500–2000	500–2500/400–2300
Vitamin E, mg/day	7 11 15	15	15	300 600 800	1000	1000	5.3–9.8/5.1–9.8 6.3–11.2/5.9–13.3 5.9–14.5/5.6–18.1 6.8–20.8/6.0–15.5	3.3–17.7/4.2–16.1	6.3–13.7/6.7–13.7
Vitamin B6, mg/day	0.6 1.0 1.3/1.2	1.3	1.7/1.5	40 60 80	100	100	1.3–1.8/1.0–1.9 1.2–2.5/1.1–1.9 1.2–2.8/1.1–2.7 1.5–3.1/1.2–2.5	1.6–3.5/1.3–2.1	1.2–3.0/1.2–2.9
Vitamin B12, μg/day	1.2 1.8 2.4	2.4	2.4	ND	ND	ND	2.7–5.3/2.6–5.0 3.6–5.5/2.2–5.3 3.2–11.8/2.2–11.1 4.9–7.5/3.5–5.2	1.9–9.3/1.0–8.8	3.1–8.2/2.5–7.5
Folate, μg/day	200 300 400	300-400	400	400 600 800	1000	1000	120–256/109–199 144–290/133–264 149–428/140–360 190–365/154–298	203–494/131–392	139–343/121–335
Zinc, mg/day	5 8 11/9	11/8	11/8	12 23 34	40	40	6.0–9.2/5.3–8.9 7.0–10.9/6.4–9.4 7.0–14.6/6.1–13.9 9.3–15.2/6.4–11.0	8.6–14.6/6.7–10.7	7.5–12.3/6.7–11.2
Iron, mg/day	10 8 11/15	8/18	8	40 40 45	45	45	7.3–10.6/6.8–10.6 8.4–11.8/7.7–11.8 9.2–19.4/7.7–14.8 10.2–19.0/7.8–14.0	10.6–26.9/8.2–22.2	10.2–25.2/8.5–20.9
Copper, μg/day	440 700 890	900	900	3000 5000 8000	10,000	10,000	700–2200/700–2000 900–2800/800–2600 800–2900/700-2800 1200–3400/800–2100	1100–2300/1000–2200	1100–1900/900–1900
Selenium, μg/day	30 40 55	55	55	150 280 400	400	400	23–61/24–61 27–41/26–58 29–110/28–104 39–59/30–38	36–73/31–54	39–62/34–55

^a^ Although adequate intake values are provided by the Institute of Medicine for infants (0–12 months) and recommended dietary allowances for children (1–3 years) [78], there are few data regarding micronutrient deficiencies in this age groups in industrialized countries and these ages have therefore not been included in this table; ^b^ values differ in pregnancy and lactation. F, females; M, males; ND, not determined.

**Table 3:** Impact of micronutrient deficiency and supplementation on immune responses and the risk of infection.

Micronutrient	Impact of Deficiency	Impact of Supplementation
Vitamin C	Increased oxidative damage [104] Increased incidence and severity of pneumonia and other infections [71,104] Decreased resistance to infection and cancer, decreased delayed-type hypersensitivity response, impaired wound healing [49]	Antioxidant properties protect leukocytes and lymphocytes from oxidative stress [14] Older people: possible reduction in incidence and duration of pneumonia [71] Children: reduced duration and severity of common cold symptoms [105]; improved outcomes in pneumonia, malaria and diarrheal symptoms [9]
Vitamin D	Increased susceptibility to infections, especially RTI [71] Increased morbidity and mortality, increased severity of infections, reduced number of lymphocytes, reduced lymphoid organ weight [49] Increased risk of autoimmune diseases (e.g., type 1 diabetes, multiple sclerosis, systemic lupus erythematosus, rheumatoid arthritis) [14]	Reduced acute respiratory tract infections if deficient [71]
Vitamin A	Affects many immune functions, including number and killing activity of NK cells, neutrophil function, macrophage ability to phagocytose pathogens, growth and differentiation of B cells, decreasing number and distribution of T cells, etc. [14] Increased susceptibility to infections (e.g., diarrhea, RTI, measles, malaria) [14,71]	Children: Reduces all-cause mortality, diarrhea incidence and mortality, and measles incidence and morbidity in deficient children (6 month to 5 years) [14,71]; decreased risk of morbidity and mortality from infectious diseases [77] Not beneficial in pneumonia [14]
Vitamin E	Deficiency rare in humans [49] Impairs both humoral and cell-mediated aspects of adaptive immunity, including B and T cell function [14]	Older people: reduced RTI [71]
Vitamin B6	Lymphocytopenia, reduced lymphoid tissue weight, reduced responses to mitogens, general deficiencies in cell-mediated immunity, lowered antibody responses [49]	
Vitamin B12	Depressed immune responses (e.g., delayed-type hypersensitivity response, T-cell proliferation) [49] *	
Folate	Depressed immune responses (e.g., delayed-type hypersensitivity response, T-cell proliferation) [49] *	
Zinc	Decreased lymphocyte number and function, particularly T cells, increased thymic atrophy, altered cytokine production that contributes to oxidative stress and inflammation [14] Increased bacterial, viral and fungal infections (particularly diarrhea and pneumonia) [71] and diarrheal and respiratory morbidity [49] Increased thymic atrophy and consequent risk of infection [97]	Restoration of thymulin activity, increased numbers of cytotoxic T cells, reduced numbers of activated T helper cells (which can contribute to autoimmunity), increased natural killer cell cytotoxicity, reduced incidence of infections [14] Children: reduction in duration of diarrhea and incidence of pneumonia in at-risk children >6 month, but not in children 2–6 month [71]; reduced duration and severity of common cold symptoms [108]; improved outcomes in pneumonia, malaria and diarrheal symptoms [9]
Iron	Reduced capacity for adequate immune response (decreased delayed-type hypersensitivity response, mitogen responsiveness, NK cell activity), decreased lymphocyte bactericidal activity, lower interleukin-6 levels [49]	May enhance or protect from infection with bacteria, viruses, fungi and protozoa depending on the level of iron [71] May theoretically enhance immunity to infectious diseases, but untargeted supplementation may increase availability of iron for pathogen growth and virulence and increase susceptibility to malaria and bacterial sepsis in particular [71] Children: potential detrimental effects in iron-replete children [14]
Copper	Abnormally low neutrophil levels [14] Potentially increased susceptibility to infection [14]	Children: increased ability of certain white blood cells to engulf pathogens if deficient [14] Reduced antibody production in response to influenza vaccine with chronic high doses in healthy young men [14]
Selenium	Impaired humoral and cell-mediated immunity [14] Increased viral virulence [14,71] Suppression of immune function, increased cancer incidence and cardiomyopathy with chronic deficiency [49] Children: increased risk of respiratory infections in the first 6 weeks of life [71]	Improves cell-mediated immunity and enhances immune response to viruses in deficient individuals, but may worsen allergic asthma and impair the immune response to parasites [14]

* Immune system effects of vitamin B12 deficiency and folate deficiency are clinically indistinguishable [49]. RTI, respiratory tract infections.
